# Vortex structure in Wigner molecules

**DOI:** 10.1038/s41598-023-36659-3

**Published:** 2023-06-15

**Authors:** Tanmay Thakur, Bartłomiej Szafran

**Affiliations:** Faculty of Physics and Applied Computer Science, AGH University, al. Mickiewicza 30, 30-059 Kraków, Poland

**Keywords:** Physics, Condensed-matter physics

## Abstract

We study clusters of vortices for Wigner molecules formed in the laboratory frame induced by anisotropy of the external potential or electron effective mass. For anisotropic systems the ground-state vortex structure undergoes a continuous evolution when the magnetic field is varied in contrast to isotropic systems where it changes rapidly at angular momentum transitions. In fractional quantum Hall conditions the additional vortices first appear on the edges of the confined system far from the axis of a linear Wigner molecule and then approach the electron positions in growing magnetic field. For an isotropic mass the vortices tend to stay at the line perpendicular to the Wigner molecule axis and pass to the axis for the lowest Landau level filling factor of $\nu \simeq \frac{1}{5}$. In phosphorene the behaviour of the vortices is influenced by a strong anisotropy of the electron effective mass. The vortices are stabilized off the axis of the molecule when it is oriented along the armchair crystal direction. For the molecule oriented along the zigzag direction the vortices are transfered to the molecule axis already at $\nu \simeq \frac{1}{3}$. The transfer is associated with an antivortex creation and annihilation near the electron position.

## Introduction

In fractional quantum Hall conditions, high magnetic field induces freezing of the kinetic energy to the lowest Landau level that results in appearance of strong electron–electron correlations. As a result of the correlations clusters of vortices appear in the ground-state wave function^1–6^. The vortices are zeroes of the wave functions accompanied by phase winding^1–9^ that are attributed to the external magnetic field flux quanta piercing the electron system^3^. Electron-vortices structures are used for construction of the composite fermion^3^ wave functions. The vortices with phase winding for the electrons at high magnetic field have their counterparts in the trapped rotating Bose–Einstein condensates^10–12^.

Structures of vortices for circular quantum dots in isotropic mass materials have been discussed for the exact diagonalization wave functions^7–9^. In addition to the formation of vortices, the electron–electron correlations lead to Wigner localization^13–16^. The finite counterparts of the Wigner crystal in laterally confined systems are called Wigner molecules^17–30^. For circularly symmetric quantum dots, Wigner molecules are formed in the inner coordinates of the system^13^ and for lowered symmetry they may appear in the laboratory frame^25–31^ with distinct single-electron islands in the charge density distribution. A lower symmetry of the system can be induced by external potential^25–31^ or by the effective mass anisotropy^32^.

The venue of new materials motivated the research on quantum Hall states^33–40^ and Wigner crystallization^32,41^ for anisotropic Fermi surface. Effects of anisotropic electron–electron interactions for quantum Hall states were also studied^42–45^. The anisotropic effective mass for a single electron can be readily accounted for in the structure of the Landau levels or Fock–Darwin states^36,46^ with rescaling the electron coordinates producing a modified angular momentum operator that commutes with the Hamiltonian. However, the central Coulomb interaction is not compatible with the rescaled coordinates and calls for further theoretical treatment of the interacting eigenstates^33–39,46^.

The purpose of the present paper is to study the fractional quantum Hall effect clusters of vortices in the presence of Wigner crystallization in the laboratory frame space induced by anisotropy of external potential or effective mass. We chose for the case study the phosphorene^47–50^ a material that exhibits a strong anisotropy of the effective mass. The quantum Hall effects in phosphorene were subject to theoretical^39^ and experimental^51–54^ investigations. Relatively large carrier effective masses in phosphorene provide favorable conditions for formation of Wigner localization, and their strong anisotropy^55–60^ paves the way for formation of Wigner molecules to be observed in the laboratory frame^32^ and not only in the inner structure of the electron system. We find that in quasi one-dimensional Wigner molecules the additional vortex clusters that appear with decreasing lowest Landau level filling factor tend to stay off the axis of the molecule for an isotropic effective mass. The anisotropy of the effective mass interferes with this process, strengthening or weakening this tendency depending on the orientation of the Wigner molecule axis with the heavier mass direction.

## Theory

We work with a two-dimensional single-electron Hamiltonian with anisotropic effective mass and parabolic confinement^61^

1 $$\begin{aligned} h= & {} {\left( -i\hbar \frac{\partial }{\partial x}+e{A_x}\right) ^2}/{2m_x} +{\left( -i\hbar \frac{\partial }{\partial y}+e{A_y}\right) ^2}/{2m_y} \nonumber \\{} & {} +\frac{1}{2}\left( m_x \omega _x^2x^2+m_y\omega _y^2y^2\right) \, \end{aligned}$$

with the symmetric gauge $\textbf{A}=(A_x,A_y,A_z)=(-By/2,Bx/2,0)$ for perpendicular magnetic field *B*. In Eq. (1) we use the effective masses^61^ for phosporene $m_x=0.17037m_0$ for the armchair crystal direction (*x*) and $m_y=0.85327m_0$ for the zigzag direction (*y*). The Hamiltonian with these mass parameters^61^ reproduces the results of the tight-binding approximation^57^ for electron states confined laterally within the monolayer phosphorene.

The single-electron wave functions $\phi$ are obtained by diagonalization of *h* in the basis of a product of polynomials and a Gaussian

2 $$\begin{aligned} \phi _\mu (\textbf{r})=\sum _\nu c^\mu _\nu x^{\nu _x}y^{\nu _y}\exp (-a_x x^2 -a_y y^2), \end{aligned}$$

where $a_x$ and $a_y$ are determined variationally and $\mu$ numbers the *h* eigenstates in the energy order.

For analysis of the zeroes in the fractional quantum Hall regime we consider a spin-polarized system and consider three electrons, which is the minimal electron number that allows for discussion of the zeroes of the reduced wave function^7–9^.

For three electrons, we use the configuration interaction approach, i.e. we build a basis of antisymmetrized products (Slater determinants) of single-electron wave functions

3 $$\begin{aligned} \Phi (\textbf{r}_1,\textbf{r}_2,\textbf{r}_3)=\sum _\eta d_\eta {\mathcal {A}} \left[ \phi _{\mu _1}(\textbf{r}_1)\phi _{\mu _2}(\textbf{r}_2)\phi _{\mu _3}(\textbf{r}_3)\right] , \end{aligned}$$

where ${\mathcal {A}}$ is the antisymmetrization operator and $\mu _1<\mu _2<\mu _3$. The expansion coefficients $d_\eta$ are obtained by diagonalization of the three-electron Hamiltonian

4 $$\begin{aligned} H=\sum _{i=1}^3 h(\textbf{r}_i)+\sum _{i=1,j>1}^3 \frac{e^2}{4\pi \epsilon \epsilon _0r_{ij}}. \end{aligned}$$

We use the dielectric constant $\epsilon =9$ for the phosphorene embedded in Al$_2$O$_3$.

The calculations for the discussion of wave function zeroes require highly convergent results. After diagonalization of the *h* operator, we use 98 lowest energy single-electron eigenstates for the construction of about 150 thousand three-electron Slater determinants that are used as a basis to provide convergence in the total energy of a fraction of a $\upmu$eV.

## Results and discussion

This section is organized as follows. We first provide the results for isotropic effective mass to set the reference for discussion of the effects of the mass anisotropy. For the isotropic mass we discuss the passage from the circular confinement to the quasi 1D confinement with the Wigner molecule in the laboratory frame. Next, we present the results for phosphorene with the Wigner molecule oriented along the zigzag or armchair crystal directions.

**Figure 1:**
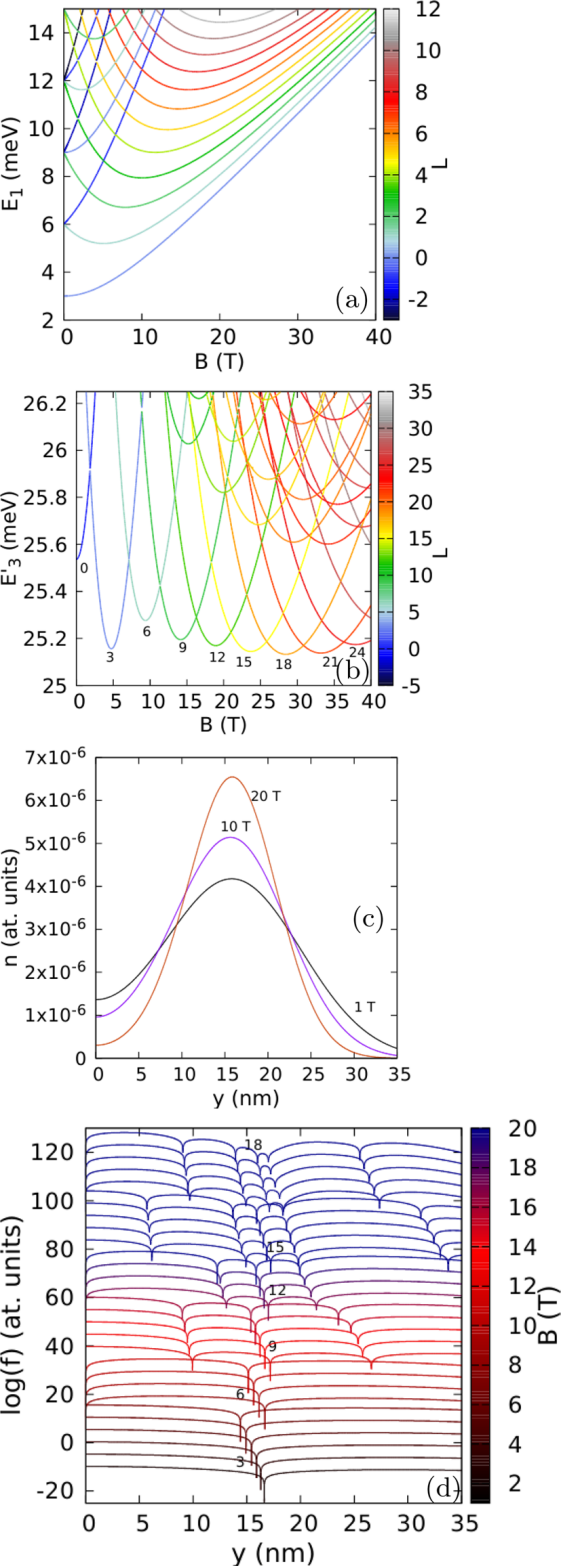
(**a**) Single-electron energy spectrum for an isotropic effective mass $m_x=m_y=0.17037m_0$ and confinement potential $\hbar \omega _x=\hbar \omega _y=3$ meV. The color of the lines corresponds to the angular momentum eigenvalue. (**b**) The three-electron energy spectrum for the spin-polarized states calculated with respect to the non-interacting ground state. (**c**) The electron density cross sections for the lowest-energy three-electron spin-polarized states density for $B=1$ T, 10 T, and 20 T. (**d**) The logarithm of the reduced wave function *f* for two electrons localized at $(x=0,y=\pm y_{max})$, where $y_{max}$ corresponds to the maximal electron density calculated along the *y* axis. The dip corresponding to the electron at $y=y_{max}$ is close to the numbers that denote the ground-state angular momentum. The plots are calculated for $B=1,2,\dots 29$ T. The lines for subsequent magnetic fields are shifted by + 5 on the plot for clarity. Here, for presentation, we plot $\log (f+10^{-15})$ instead of $\log (f)$ to make the dips of the wave function shallower.

**Figure 2:**
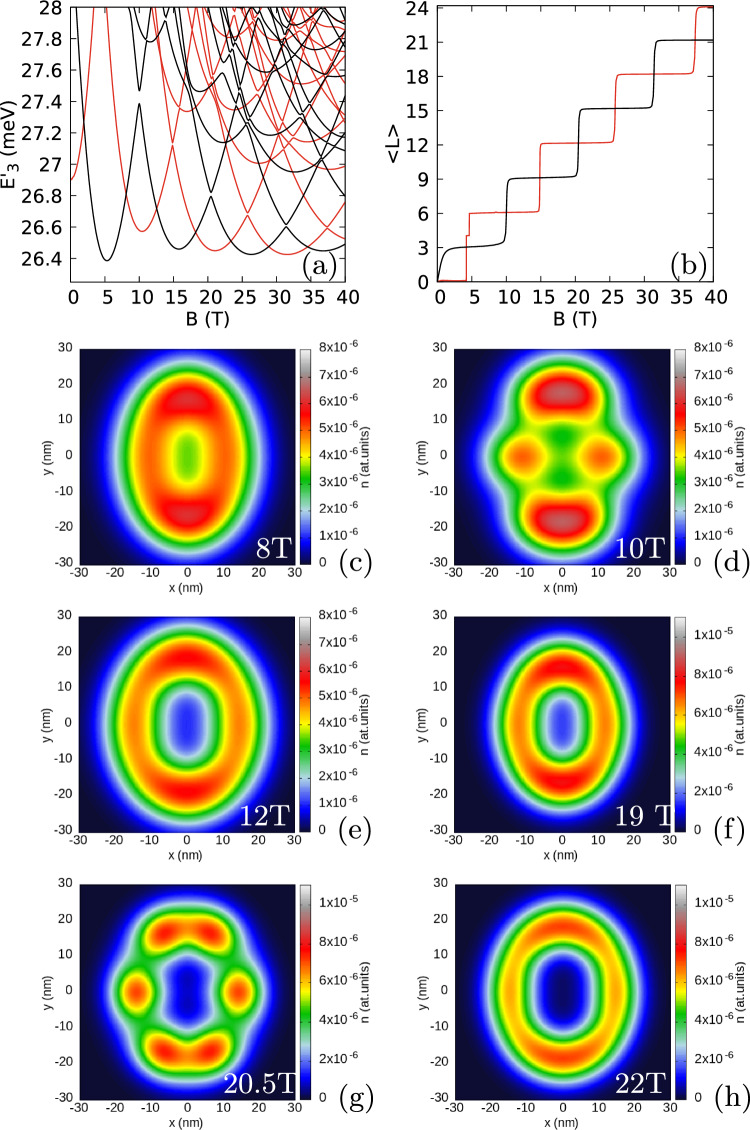
Results for an isotropic electron mass $m_x=m_y=0.17037m_0$ with a weak anisotropy of the confinement potential $\hbar \omega _x=3.5$ meV and $\hbar \omega _y=3$ meV. (**a**) The energy spectrum for spin-polarized three-electron states minus three times the single-electron ground state energy. (**b**) The average value of the angular momentum for the lowest-energy odd and even parity states. In (**a**) and (**b**) the values for odd parity states are plotted in black and for the even parity ones in red. (**c**–**h**) The electron density for the lowest-energy odd parity states.

**Figure 3:**
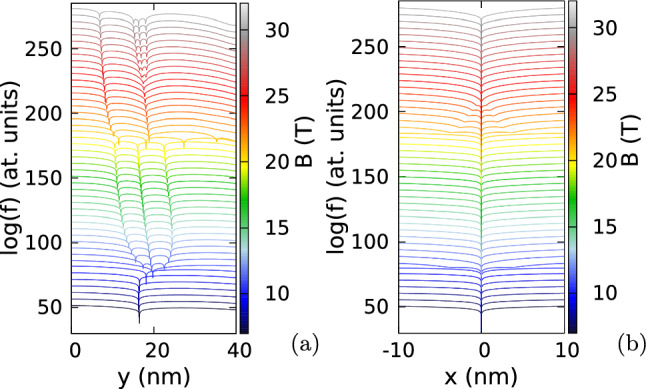
Logarithm of the reduced wave function for the lowest-energy odd-parity state for $m_x=m_y=0.17037m_0$, $\hbar \omega _x=3.5$ meV and $\hbar \omega _y=3$ meV (as in Fig. 2) for two electrons localized at $(x=0,y=\pm y_{max})$, where $y_{max}$ corresponds to the maximal electron density calculated along the *y* axis. (**a**) Shows the cross section along the *y* axis, and (**b**) the cross section along the *x* direction for $y=y_{max}$. The plots are calculated for $B=7$ T do 30 T with steps of 0.5 T. The lines for subsequent magnetic fields are shifted by + 10 on the plot for clarity. In (**a**) we plot $\log (f+10^{-14})$ instead of $\log (f)$ to make the dips of the wave function shallower. The snapshots of the reduced wave function on (*x*, *y*) plane for selected values of the magnetic field are given in the Supplementary information (Supplementary Fig. S1). The dips in (**b**) are shallower since the zeroes that appear off the *y* axis are displaced along the *y* axis by an amount that changes with *B* (see Supplementary Fig. S1 in the Supplementary information).

### Isotropic effective mass

The results for a circular potential $\hbar \omega =\hbar \omega _x=\hbar \omega _y=3$ meV with an isotropic effective mass $m=m_x=m_y=0.17037m_0$ are summarized in Fig. 1. At high magnetic field the single-electron energy levels with non-negative angular momenta form a band that tends to the lowest Landau level (Fig. 1a). The three-electron spectrum presented in Fig. 1b exhibits the angular momentum transitions in the ground state^13^. The spin-polarized ground-state angular-momentum quantum numbers *L* take values that are multiples of 3^9^. The eigenvalue of the spatial parity operator for a given *L* is $(-1)^L$. In the following, we will refer to the states with the negative (positive) eigenvalue of the spatial parity as odd (even) parity states. The Laughlin wave function^1^—in form of the antisymmetric Jastrow factor attenuated by a Gaussian—provides an approximate eigenstates for the ground states of odd parity. For the three-electron state at the lowest Landau level filling factor of $\nu =\frac{1}{n}$ with odd *n* this wave function has the form

5 $$\begin{aligned} \Psi _{\nu =\frac{1}{n}}(z_1,z_2,z_3)= & {} \exp (-\frac{|z_1|^2+|z_2|^2+|z_3|^2}{{2l_B^2}}) \nonumber \\{} & {} \quad \times \left[ (z_1-z_2)(z_2-z_3)(z_1-z_3)\right] ^n, \end{aligned}$$

where $z=x+iy$, $l_B^2=\frac{\hbar }{m \omega _h}$, with $\omega _h^2=\omega ^2+\omega _c^2/4$ and the cyclotron frequency $\omega _c=\frac{eB}{m}$. This wave function is an eigenstate of the angular momentum for $L=3n$, hence the relation between the angular momentum and the lowest Landau level filling factor $\nu =\frac{3}{L}$^7–9^.

In the following we discuss the position of vortices of the reduced wave function^7,8^, e.g. the three-electron wave function for two fixed electron positions

6 $$\begin{aligned} g(z;z_1,z_2)\equiv \Phi (z_1,z_2,z). \end{aligned}$$

The reduced Laughlin wave function is a complex polynomial of degree $2n=\frac{2}{\nu }=\frac{2}{3}L$, that possesses two *n*-fold zeroes at the electron positions: one at $z=z_1$ and the other at $z=z_2$. In the context of phase circulation, these zeroes are giant vortices with the phase of *g* that changes by $2\pi n$ under a rotation around the zero.

The Laughlin wave function is the exact^2^ solution for the contact electron–electron interaction potential within the lowest Landau level. For the Coulomb interaction potential $2n-2$ zeroes detach from the fixed electron positions^7,8^ and the zeroes are single vortices with rotation of the phase by $2\pi$. In Fig. 1d we plot the logarithm of the absolute value of the reduced wave function $f(z)=|g(z;z_1,z_2)|$ with one electron fixed at $(0,y_{max})$ and the other at $(0,-y_{max})$, where $y_{max}$ corresponds to the maximum of the electron density (Fig. 1c). The value of $y_{max}$ decreases with increasing *B* for fixed value of the angular momentum, and increases in a discontinuous manner once a larger *L* appears in the ground state at an angular momentum transition. In Fig. 1d, $y_{max}$ corresponds to the central dip of the reduced wave function. We denote the ground-state angular momentum with integer values placed near zero at $y=y_{max}$ in the figure. For $L=3$, the reduced wave function has only the zeroes that correspond to the fixed positions of electrons. The next ground state at higher field, with $L=6$ is of the even spatial parity and does not correspond to the Laughlin wave function. For $L=9$ and the filling factor $\nu =\frac{1}{3}$—covered with a Laughlin wave function—the number of zeroes is 6, e.g. three per fixed electron. We find that the extra zeroes appear also on the *y* axis, one closer and one further apart from the potential center than the electron position. The state with $L=12$ is a non-Laughlin state with an extra zero in the confinement potential center. For $L=15$ (Laughlin filling factor $\nu =\frac{1}{5}$) we find 5 zeroes per fixed electron position, etc.

Generally, for the state of the total angular momentum *L* diagonalized on the lowest Landau level basis with single-electron states of non-negative angular momenta $L=L_1+L_2+L_3$, the maximal degree of the reduced wave function is $L-1$ (for the minimal value of a sum $L_1+L_2=1$). Therefore, for $L=15$ one could expect up to 7 zeroes per fixed electron and not 5 as in the Laughlin wave function. Since our calculation is not limited to the lowest Landau level and covers also the states with negative single-electron angular momenta, the maximal degree of the polynomial, and the number of zeroes of the reduced wave function could be even larger, but for the circular quantum dot in the Lauglin state we resolve only the number of zeroes expected for the Laughlin wave function, i.e. *L*/3 or *n* per electron. More zeroes may still appear in the region far away from the dots center, where the electron density is negligible. The presence and location of zeroes far away from the region occupied by the electron density have no significant effect for the system energy.

For confinement that deviates from the circular symmetry we find a mechanism of formation and annihilation of antivortices, when the number of zeroes changes at a small magnetic field variation. To show this effect, let us now consider a deviation from the circular symmetry of the confinement potential. We increase the confinement energy in the *x* direction to $\hbar \omega _x=3.5$ meV. The three-electron spectrum (Fig. 2a) can no longer be described by the angular momentum, but the spatial parity is still defined for the Hamiltonian eigenstates. The quantum mechanical expectation value of the angular momentum which is plotted in Fig. 2b possesses plateaux near even and odd integer values for even and odd parity states, respectively. The steps between the plateaux correspond to avoided crossings that open between the lowest- and second-energy states of the same parity (Fig. 2a). The electron density on the plateaux [(Fig. 2c,e,f,h) for odd-parity states near $\langle L\rangle =3$, 9 and 15] has the form of an elliptically deformed ring. At the steps between the plateaux (Fig. 2d,g) local maxima appear in the density. These densities are superpositions of two energy-equivalent semiclassical^62^ charge configurations with the central electron occupying one of the maxima at the *x* axis.

**Figure 4:**
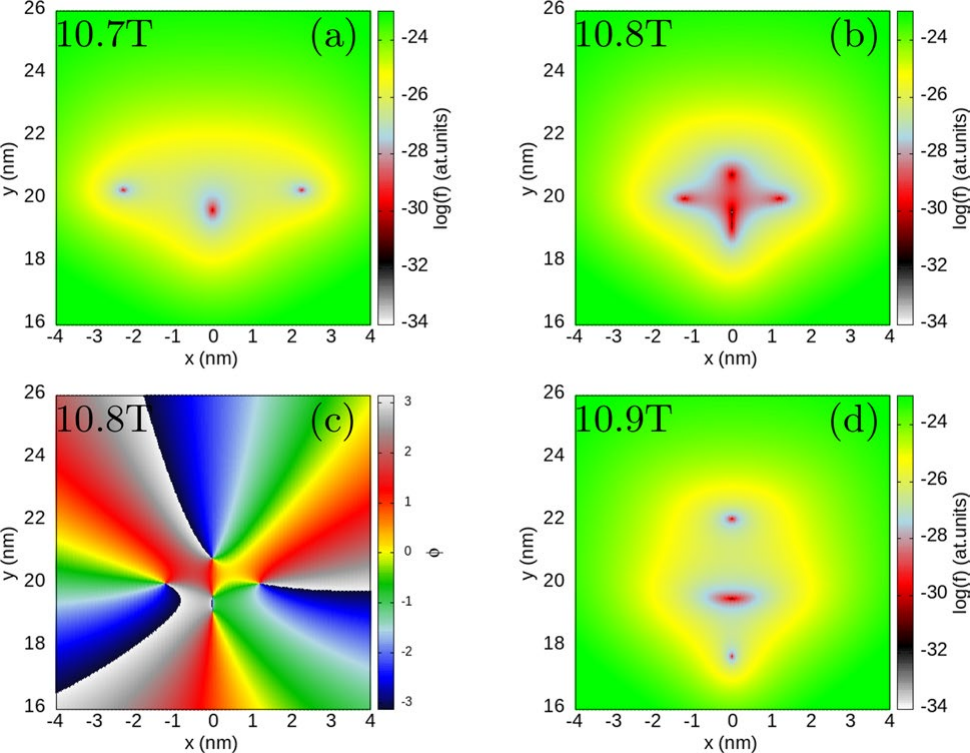
Logarithm of the reduced wave function for the lowest-energy odd-parity state for $m_x=m_y=0.17037m_0$, $\hbar \omega _x=3.5$ meV and $\hbar \omega _y=3$ meV as in Fig. 2. Two electrons are fixed at $(0, \pm y_{max})$, where $y_{max}$ corresponds to the maximal value of the electron density for $B=10.7$ T (**a**), 10.8 T (**b**) and 10.9 T (**d**), respectively. In (**c**) we plot the phase of the reduced wave function for $B=10.8$ T.

**Figure 5:**
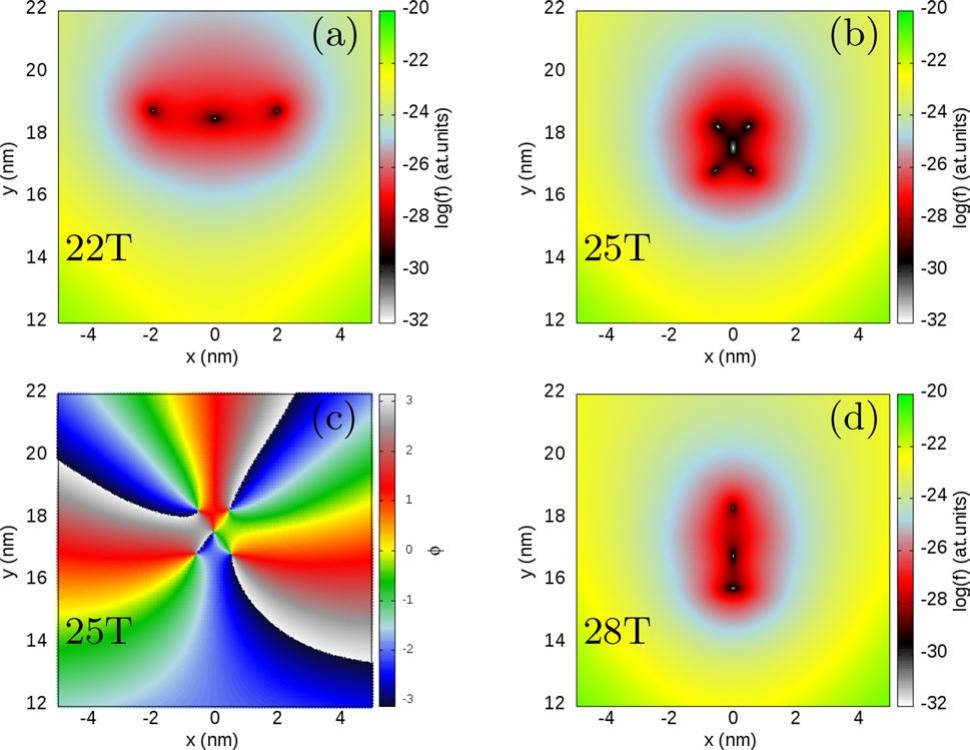
(**a**,**b**,**d**) same as Fig. 4 only for $B=22$ T, 25 T and 28 T, respectively. In (**c**) we plot the phase of the reduced wave function for $B=25$ T.

In Figs. 3, 4 and 5 we look at detailed behaviour of the zeroes of the reduced wave function of the lowest-energy odd state (see also Supplementary Fig. S1 in the Supplementary Information) in the region of non-zero charge density. Below 10 T—at the plateaux of $\langle L\rangle \simeq 3$—the zeroes appear only at the fixed electron positions. At the step between the plateaux with average *L* of 3 and 9 two additional zeroes appear on the edges of the reduced wave function [see the dips for 10.5 T in Fig. 3b]. These zeroes approach the fixed electron position as *B* grows. At 12 T already at the plateaux of $\langle L\rangle \simeq 9$ the additional zeroes appear at the *y* axis (Fig. 3a) as for the circular quantum dot (cf. Fig. 1d). The mechanism of the transition of the zeroes to the *y* axis is presented in Fig. 4. Between 10.7 T and 10.8 T, the zero associated with the electron position is split into three zeroes, all aligned at the *y* axis. In Fig. 4b the zero at the electron position is the central one in the cross configuration. The phase of the reduced wave function plotted in Fig. 4c shows that four of the zeroes at the arms of the cross are associated with a growth of the phase in the clockwise rotation around the zeroes. Only the phase at the central zero decreases in the clockwise direction. In this sense, splitting of the zero at the fixed electron position corresponds to transformation of a single vortex (Fig. 4a) to a central antivortex and two vortices above and below the antivortex (Fig. 4b,c). As *B* is increased further (Fig. 4d) the additional vortices at the *y* axis go further away from the electron position and the two at the sides approach the central antivortex. Due to the vortex-antivortex annihilation we are left with a single vortex at the electron position.

The reduced wave function for $B=20.5$ T (Fig. 3b), that is, at the step between the plateaux with $\langle L\rangle$ of 9 and 15 (Fig. 2b) another two extra zeroes appear at a distance from the *y* axis. These zeroes approach the fixed electron position as *B* grows (Fig. 3b) and the highest one on the *y* axis moves away from the electron position (Fig. 3a) and leaves the picture. Between 22 T and 28 T the zeroes on the sides of the system relocate to the *y* axis with a process involving the formation of an antivortex at the electron position (Fig. 5b,c) and two additional vortices and next annihilation of the two vortices that approach the electron position along the *x* axis with the antivortex at $z_1$ (Fig. 5d). This process is similar to the one seen above in Fig. 4.

Note that for a circular system and a state of a fixed angular momentum, the variation of the magnetic field reduces to scaling the electron coordinates^63^ accompanied by a scaling of the positions of the vortices. For systems with broken rotational symmetry we find a continuous evolution of the geometry of the vortex cluster beyond a simple scaling.

**Figure 6:**
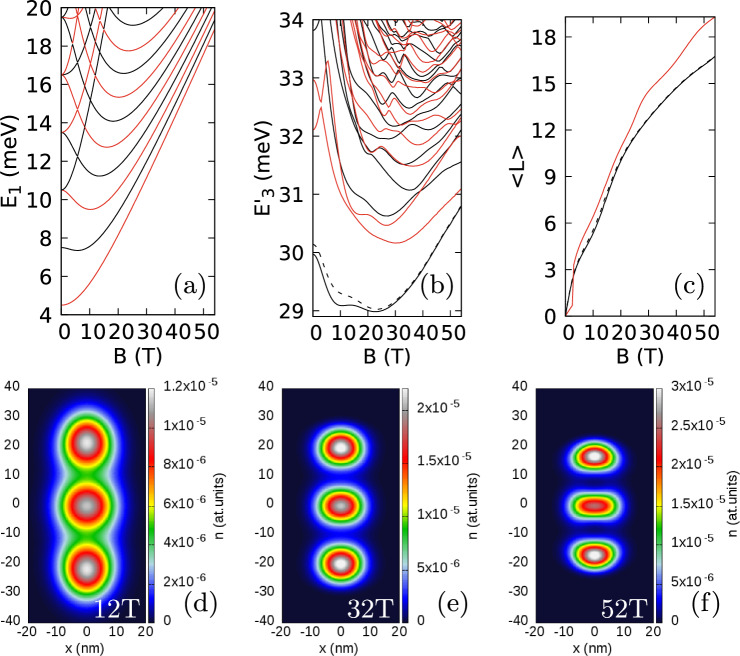
Results for an isotropic electron mass $m_x=m_y=0.17037m_0$ with a strong anisotropy of the confinement potential $\hbar \omega _x=6$ meV and $\hbar \omega _y=3$ meV. (**a**) Single electron spectrum. (**b**) The energy spectrum for three electrons minus three times the single-electron ground state energy. (**c**) Average angular momentum of the three electron system. In (**a**–**c**) the values for the even (odd) parity states are plotted in red (black). In (**b**) and (**c**) the dashed black line shows the results for the ground-state calculated in the basis limited to the non-negative average value of the angular momentum. (**d**,**e**,**f**) The electron densities at 12 T, 32 T, and 52 T, respectively.

Let us now look at the system with strong anisotropy of the external potential ($\hbar \omega _x=6$ meV and $\hbar \omega _y=3$ meV). The three-electron ground state is odd in the spatial parity (Fig. 6b) for any *B* and the spacings between the ground state and the higher energy levels are too wide for the avoided crossings to be resolved. The dependence of the average angular momentum on *B* is smooth (Fig. 6c). The charge density is organized in a linear Wigner molecule (Fig. 6d–f) with single-electron islands that are increasingly localized in growing magnetic field.

**Figure 7:**
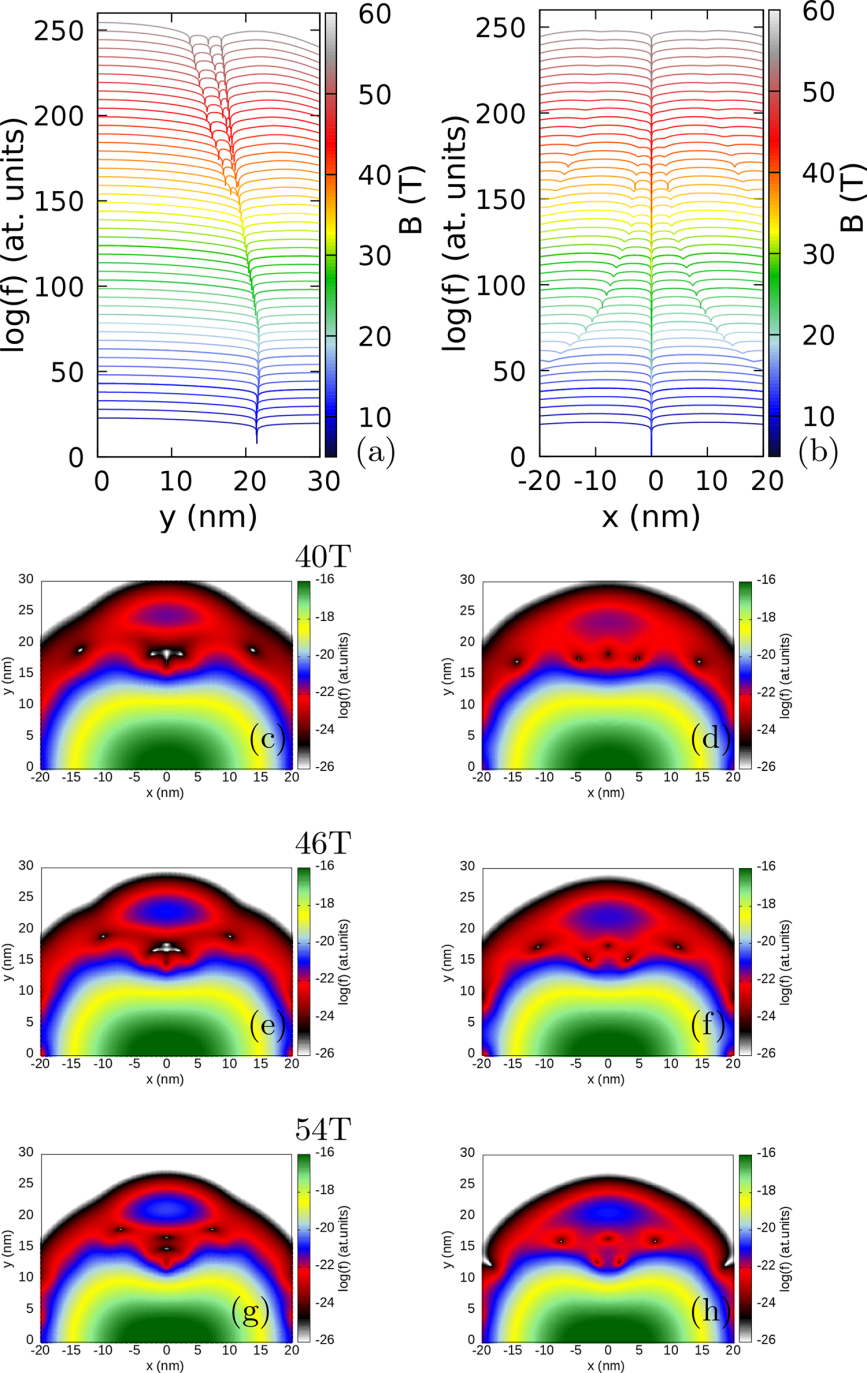
Logarithm of the reduced wave function for the lowest-energy odd-parity state for $m_x=m_y=0.17037m_0$, $\hbar \omega _x=6$ meV and $\hbar \omega _y =3$ meV as in Fig. 6. In the plots two electrons are localized at positions $(x=0,y=y_{max})$ where $y_{max}$ corresponds to the maximal electron density calculated along the *y* axis. (**a**) Shows the cross section along the *y* axis and (**b**) the cross section along the *x* direction for $y=y_{max}$. The lines are plotted from 8 T to 54 T with steps of 1 T. In (**a**,**b**) the lines for subsequent magnetic fields are shifted by + 5 on the plot for clarity. In (**a**) $\log (f+10^{-14})$ is plotted instead of $\log (f)$ to make the dips of the wave function shallower. In (**c**–**h**) we plot the reduced wave function on the (*x*, *y*) plane to illustrate the behaviour of the vortices which move away from the $y=y_{max}$ position at higher B. The left column of plots—the complete basis as everywhere else in this paper. The right column of plots—the basis limited to the single-electron states with non-negative average angular momenta (the energy level marked with the dashed line in Fig. 6). Each row of plots corresponds to the same value of the magnetic field. The vortex corresponding to the electron at $(0,y_{max})$ is the one at the most central position on the *y* axis. See also Supplementary Fig. S2 for the maps at lower magnetic fields.

For 8 T, the zeroes are observed only at the fixed electron position both in the exact calculation (Fig. 7a,b). Additional zeroes appear at the edges of the plot for 16 T (Fig. 7b) and approach the zero associated with the localized electron for higher field (Fig. 7b). At 34 T (Fig. 7b) two new zeroes appear at the sides of the Wigner molecule, while the old ones remain aligned perpendicular to the orientation of the Wigner molecule. At still higher magnetic field (Fig. 7c–h) we see (Fig. 7c,e,g) the flip of two zeroes close to the electron to the axis of the Wigner molecule from the perpendicular alignment, which appears in the mechanism involving formation and annihilation of the antivortex as seen above.

In Fig. 6b,c we additionally plotted with the dashed lines the results obtained for the ground state with a basis limited to single-electron states with non-negative angular momentum. In the context of the number of zeroes of the reduced wave function the limited basis is equivalent to the lowest Landau-level approximation. Although at a high magnetic field the energies are similar on the scale of the plot, the differences are resolved on the structure of the reduced wave function zeroes (Fig. 7c–h). In the limited basis (Fig. 7d,f,h) the antivortex is not formed, and the extra zeroes accumulate around the electron position, but do not approach it as close as in the exact calculation (Fig. 7c,e,g).

**Figure 8:**
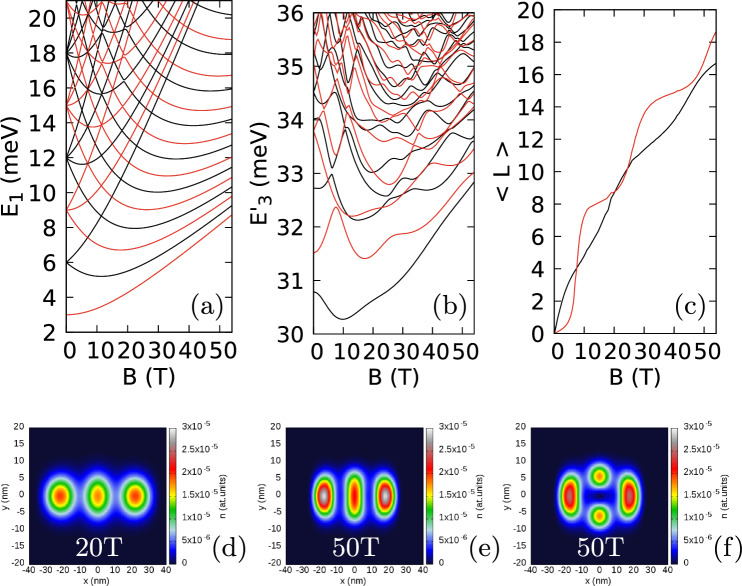
Results for the phosphorene $m_x=0.17037m_0$, $m_y=0.85327m_0$ for confinement potential with equal oscillator energies $\hbar \omega _x=\hbar \omega _y=3$ meV. (**a**) The single-electron energy levels. (**b**) The three-electron spectrum minus three times the single-electron ground-state energy. In (**a**–**b**) the red (black) color stands for the even (odd) parity states. (**c**) The average angular momentum for the even (red) and odd (black) parity energy level. (**d**,**e**) The ground electron density for 20 and 50 T, respectively. (**f**) The electron density for the lowest even-parity state at $B=50$ T.

**Figure 9:**
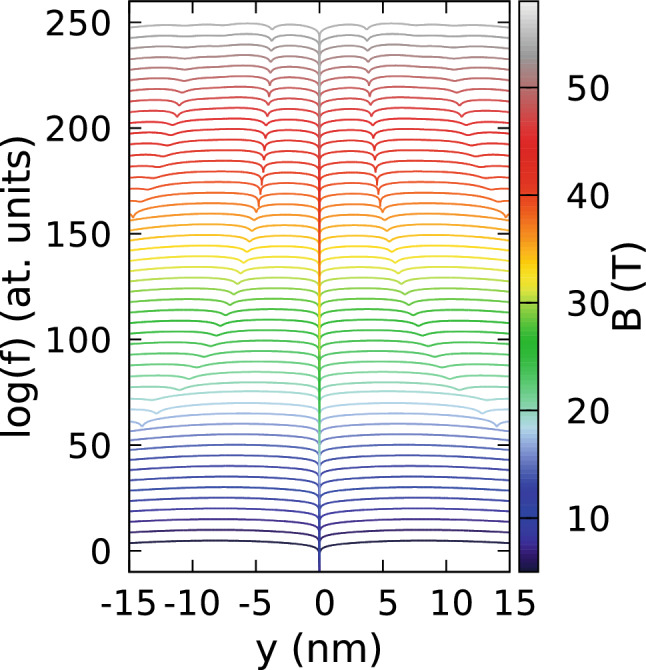
Logarithm of the reduced wave function for the lowest-energy odd-parity state for phosphorene $m_x=0.17037m_0$, $m_y=0.85327m_0$ and equal oscillator energies $\hbar \omega _x=\hbar \omega _y=3$ meV (the same parameters as in Fig. 8). Two electrons are fixed at $(0,\pm x_{max})$ points where $x_{max}$ corresponds to the maximal value of the electron density. The vortex corresponding to the electron at $(0,x_{max})$ is the one at the most central position on the *x* axis. The lines show the cross section along *y* direction for $x=x_{max}$ and are plotted from 5 T to 54 T with steps of 1 T. Each line for growing magnetic field is shifted along the vertical axis by + 5. $x_{max}$ stands for the location of the maximal charge density of the extreme charge islands—see Fig. 8e,d. Maps at (*x*, *y*) plane for selected magnetic fields are given in Supplementary Fig. S3 in the Supplementary information.

**Figure 10:**
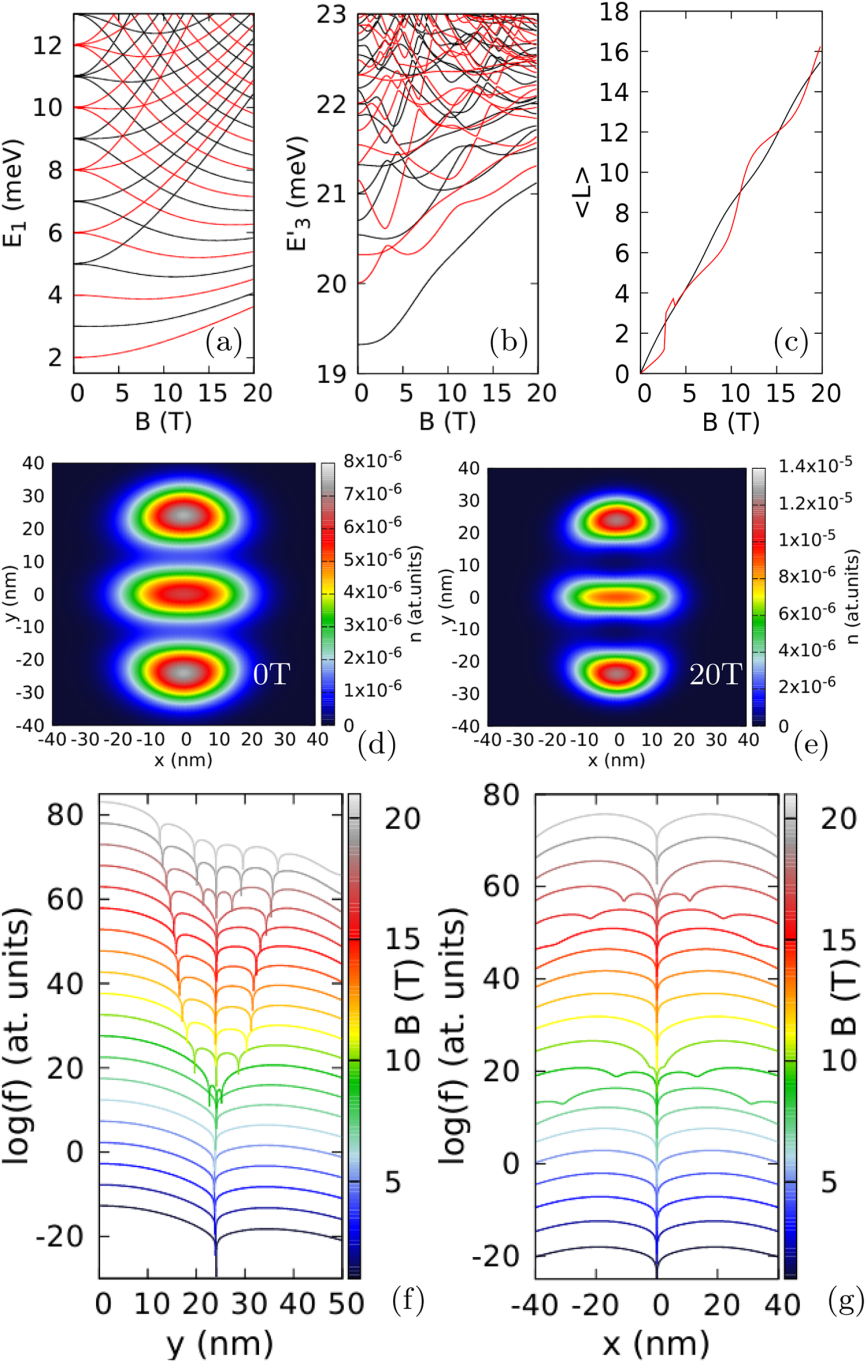
Results for the phosphorene $m_x=0.17037m_0$, $m_y=0.85327m_0$ for $\hbar \omega _x=3$ meV and $\hbar \omega _y=1$ meV. (**a**) The single-electron energy levels. (**b**) The three-electron spectrum minus three times the single-electron ground-state energy. In (**a**,**b**) the red (black) color stands for the even (odd) parity states. (**c**) The average angular momentum for the even (red) and odd (black) parity energy level. (**d**,**e**) The ground electron density for 0 and 20 T, respectively. (**f**,**g**) Logarithm of the reduced wave function for the lowest-energy odd-parity state for phosphorene—cross sections along the *y* (**f**) and *x* axis (**g**). Two electrons are fixed at $(0,\pm y_{max})$ points where $x_{yax}$ corresponds to the maximal value of the electron density. The vortex corresponding to the electron at $(0,y_{max})$ is the one at the most central position on the *y* axis. The plots are calculated from 1 T to 20 T with steps of 1 T. The values for each subsequent *B* is shifted up by 5 along the scale.

### Phosphorene: anisotropic effective mass

We now adopt the phosphorene parameters for the anisotropic electron mass $m_x=0.17037 m_0$ and $m_y=0.85327m_0$ (Fig. 8). For equal oscillator confinement energies $\hbar \omega _x=\hbar \omega _y=3$ meV (Fig. 8) the Wigner molecular form of the density appears along the *x* axis due to the large mass in the *y* direction (Fig. 8d,e). The three-electron spectrum and the dependence of the angular momentum on *B* is similar to the one for the isotropic effective mass (Fig. 6b,c): the ground state has the odd spatial parity for all *B* and the angular momentum for the ground state is a smooth function of *B* with no clearly defined plateaux. The structure of zeroes is shown in Fig. 9. The extra zeroes appear and stay near the line perpendicular to the axis of the Wigner molecule for the entire considered magnetic field range. No passage to the axis of the molecule is found, as in the case with isotropic electron mass (Fig. 7a,c,e,g). For the maximal magnetic field considered (54 T) the ground state average angular momentum exceeds $16{\hbar }$ (Fig. 8c). For the isotropic mass, the passage of the zeroes from the axis perpendicular to the molecule axis to this axis appeared for $\langle L\rangle$ was around 15 (Figs. 6c, 7c,e,g) for 5 vortices per electron only for $\nu \simeq \frac{1}{5}$.

To separate the effect of the anisotropy of the Wigner molecule from the anisotropy of the mass, we have produced the Wigner molecule oriented along the *y* axis by decreasing the confinement energy $\hbar \omega _y$ to 1 meV (Fig. 10). The character of the spectrum and angular momentum dependence on *B* remains similar to that for the molecule oriented along the *x* axis, but the zeroes structure is very different. In Fig. 10f,g we have plotted the cross sections of the reduced wave function along the axis of the molecule (Fig. 10f) and a line perpendicular to the axis along the *x* direction for $y=y_{max}$. Now, the axis of the molecule is aligned with the direction of the heavier mass. The zeroes come from the sides of the main zero (Fig. 10g—the first extra two for 8 T and 9 T, and the next extra pair for 16 T and 17 T), but get aligned with the *y* axis (Fig. 10f). The behaviour of the zeroes in Fig. 10f,g resemble rather the case of a weakly anisotropic confinement for isotropic effective mass (Fig. 3) than the results for the Wigner molecule presented above.

The tendency of the vortices to stay at the line perpendicular to the Wigner molecule was present for the isotropic mass (Fig. 7). This tendency is strengthened for the Wigner molecule with the anisotropic effective mass when the axis of the molecule is perpendicular to the direction of the heavier mass (Fig. 9) and weakened or nearly lifted (Fig. 10f,g) for the axis of the Wigner molecule aligned with the direction of the heavier mass.

The strong tendency of the vortices to be repelled from the Wigner molecule axis when it is aligned with the lighter magnetic field (Figs. 8 and 9) can be understood in the following manner. The effect is observed for both the exact calculation and in the limited basis corresponding to the lowest Landau level approximation (Fig. 7c–h). Diagonalization of the three-electron Hamiltonian in the basis of the lowest Landau level is equivalent to the diagonalization of the Coulomb interaction only, since all the states correspond to the same kinetic-energy^7,8^. Within the quasi-1D Wigner molecule the electrons are ordered along its axis in a way that does not essentially change with the magnetic field. Incorporation of the vortices to the axis of the molecule affect the electron distribution in the inner coordinates of the system. The variation of the wave function in the direction of the lighter electron mass is associated with a larger increase in the kinetic energy. When the axis of the Wigner molecule coincides with the lighter electron mass the basis of the accessible low energy states does not allow the relocation of the vortices to the axis, while it is still possible for the orthogonal orientation of the molecule (Figs. 6, 7).

The charge density distribution in Wigner phase can be studied using the scanning probe techniques^14^. However, the location of the vortices is a feature of the wave function that is not directly accessible to the experiment. The number of vortices per electron is deduced from conductance plateaux in experiments on electron gas in the fractional quantum Hall conditions. For systems confined in the quantum dots the quantity that is readily derived from the Coulomb blockade spectroscopy is the energy of the system. This experimental technique provides resolution of the energy with a precision below 10 $\upmu$eV at temperature of 0.1 K^64^. The positions of the vortices are relevant for the energy. The exact diagonalization results of this work provide information for modeling with simpler variational wave functions.

At large magnetic fields the odd-parity ground-state is separated by an energy gap of only about 0.25 meV from the first excited state which is of an even parity (see Fig. 6b or Fig. 8b at the end of the magnetic field scale. Small spacings between the energy levels is used as a signature of the Wigner physics^30^. The gap corresponds to a thermal excitation of about 2.9 K. For a strictly 1D quantum wires a thermal enhancement of Wigner oscillations in a range of temperatures at the onset of the formation of a Wigner molecule was reported^65^. The present system is not strictly 1D. In Fig. 8f we plotted the charge density of the lowest excited state. The central single-electron island is split in two, so one should expect that the thermal excitations will in our case rather destablize the charge density in terms of islands containing a single electron each. However, as pointed above experiments can be carried out at temperatures as low as 0.1 K^64^.

## Summary and conclusions

We have discussed the vortex clusters *for* Wigner molecules induced by anisotropy of confinement and anisotropy of the effective mass using an exact diagonalization approach. The ground states in one-dimensional Wigner molecules possess negative spatial parity as the Laughlin wave function. The observed number of vortices per electron for average angular momentum agrees with the ones predicted by the Laughlin function. As the magnetic field and average angular momentum increase, additional vortices appear from the lateral sides of the Wigner molecule and next approach the electron position. For the anisotropy of confinement alone, the vortex clusters tend to appear off the axis of the molecule and pass to the axis only for filling factor of $\nu \simeq \frac{1}{5}$. When the axis of the molecule coincides with the lighter hole mass, the additional vortices are stabilized off the Wigner molecule *axis*. On the contrary, for the Wigner molecule axis aligned with the heavier mass direction, the vortices are transferred to the axis of the molecule already at $\nu \simeq \frac{1}{3}$. The transfer of vortices from the perpendicular to the parallel to the axis of the molecule is accompanied with antivortex formation and annihilation that can be described only with the basis not limited to the lowest Landau level.

## Supplementary Information

Supplementary Information.

## Data Availability

The data that support the findings of this study are available from the first author (T.T.) upon reasonable request.
