# Supplementary material for: Vortex structure in Wigner molecules
Source: Sci Rep. 2023 Jun 15;13:9707. doi: 10.1038/s41598-023-36659-3 (PMC10272190; doi:10.1038/s41598-023-36659-3)
Supplement: Supplementary file 1 — Supplementary Information. [file 41598_2023_36659_MOESM1_ESM.pdf]

Vortex structure in Wigner molecules: Supplementary Information

Tanmay Thakur and Bartłomiej Szafran*
AGH University,
Faculty of Physics and Applied Computer Science,
al. Mickiewicza 30, 30-059 Kraków, Poland
**corresponding author: bszafran@agh.edu.pl*

In this supplementary information, we show the snaphsots of the logarithm reduced wave function plotted in the (x, y) plane for selected magnetic fields to complete the information given in the cross sections in the main body of the text.

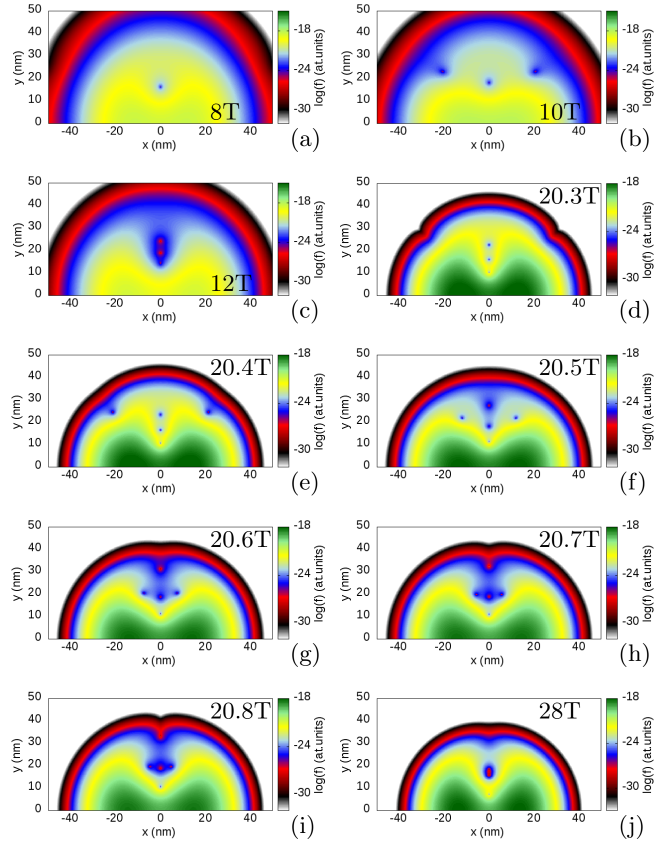

FIG. S1. Logarithm of the reduced wave function for the lowest-energy odd-parity state for $m_x = m_y = 0.17037m_0$, $\hbar\omega_x = 3.5$ meV and $\hbar\omega_y = 3$ meV as in Fig. 2 of the Article. Two electrons are fixed at $(0, \pm y_{max})$ points where y_{max} corresponds to the maximal value of the electron density. The cross sections of the logarithm of the reduced wave function are plotted in Fig. 3 of the Article.

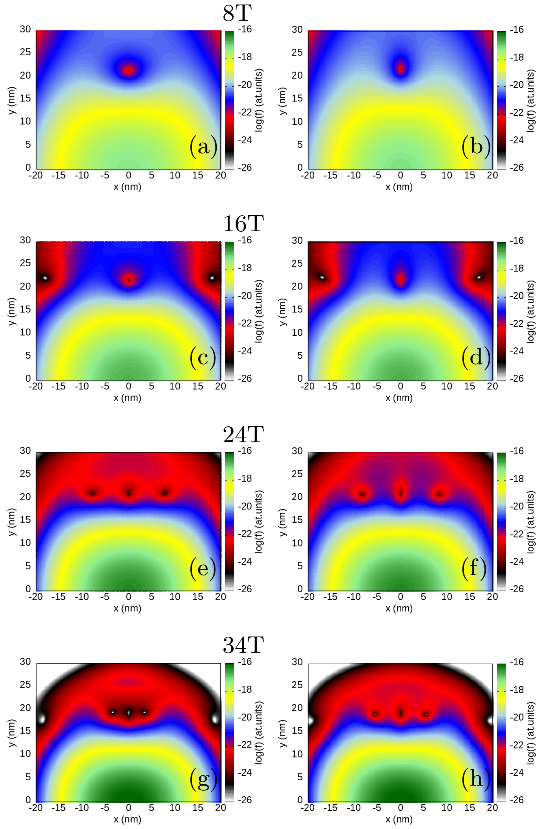

FIG. S2. Logarithm of the reduced wave function for the lowest-energy odd-parity state for $m_x = m_y = 0.17037m_0$, $\hbar\omega_x = 6$ meV and $\hbar\omega_y = 3$ meV as in Fig. 6 and Fig. 7 in the main text of the Article. Two electrons are fixed at $(0, \pm y_{max})$ points where y_{max} corresponds to the maximal value of the electron density. The left column of plots – the complete basis. The right column of plots – the basis limited to the single-electron states with non-negative average angular momenta (the energy level marked with the dashed line in Fig. 6 in the Article). Each row of plots corresponds to the same value of the magnetic field. The vortex corresponding to the electron at $(0, y_{max})$ is the one at the most central position on the y axis.

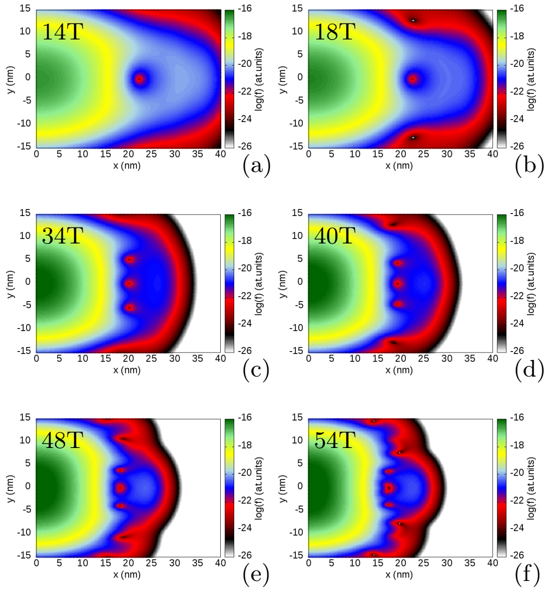

FIG. S3. Logarithm of the reduced wave function for the lowest-energy odd-parity state for phosphorene $m_x = 0.17037m_0$, $m_y = 0.85327m_0$ and equal oscillator energies $\hbar\omega_x = \hbar\omega_y = 3$ meV (the same parameters as in Figs. 8 and 9 of the Article). Two electrons are fixed at $(0, \pm x_{max})$ points where x_{max} corresponds to the maximal value of the electron density. The vortex corresponding to the electron at $(0, x_{max})$ is the one at the most central position on the x axis.
